# Impact of *ABCG2* and *ABCB1* Polymorphisms on Imatinib Plasmatic Exposure: An Original Work and Meta-Analysis

**DOI:** 10.3390/ijms24043303

**Published:** 2023-02-07

**Authors:** Chiara Dalle Fratte, Jerry Polesel, Sara Gagno, Bianca Posocco, Elena De Mattia, Rossana Roncato, Marco Orleni, Fabio Puglisi, Michela Guardascione, Angela Buonadonna, Giuseppe Toffoli, Erika Cecchin

**Affiliations:** 1 Experimental and Clinical Pharmacology, Centro di Riferimento Oncologico di Aviano (CRO), IRCCS, 33081 Aviano, Italy; 2 Unit of Cancer Epidemiology, Centro di Riferimento Oncologico di Aviano (CRO), IRCCS, 33081 Aviano, Italy; 3 Doctoral School in Pharmacological Sciences, University of Padua, 35131 Padova, Italy; 4 Department of Medical Oncology, Unit of Medical Oncology and Cancer Prevention, Centro di Riferimento Oncologico di Aviano (CRO), IRCCS, 33081 Aviano, Italy; 5 Department of Medicine, University of Udine, 33100 Udine, Italy

**Keywords:** imatinib mesylate, ABCB1, ABCG2, pharmacogenetics, therapeutic drug monitoring, GIST, CML

## Abstract

Adequate imatinib plasma levels are necessary to guarantee an efficacious and safe treatment in gastrointestinal stromal tumor (GIST) and chronic myeloid leukemia (CML) patients. Imatinib is a substrate of the drug transporters ATP-binding cassette subfamily B member 1 (ABCB1) and ATP-binding cassette subfamily G member 2 (ABCG2) that can affect its plasma concentration. In the present study, the association between three genetic polymorphisms in *ABCB1* (rs1045642, rs2032582, rs1128503) and one in *ABCG2* (rs2231142) and the imatinib plasma trough concentration (C_trough_) was investigated in 33 GIST patients enrolled in a prospective clinical trial. The results of the study were meta-analyzed with those of other seven studies (including a total of 649 patients) selected from the literature through a systematic review process. The *ABCG2* c.421C>A genotype demonstrated, in our cohort of patients, a borderline association with imatinib plasma trough levels that became significant in the meta-analysis. Specifically, homozygous carriers of the *ABCG2* c.421 A allele showed higher imatinib plasma C_trough_ with respect to the CC/CA carriers (C_trough_, 1463.2 ng/mL AA, vs. 1196.6 ng/mL CC + AC, *p* = 0.04) in 293 patients eligible for the evaluation of this polymorphism in the meta-analysis. The results remained significant under the additive model. No significant association could be described between *ABCB1* polymorphisms and imatinib C_trough_, neither in our cohort nor in the meta-analysis. In conclusion, our results and the available literature studies sustain an association between *ABCG2* c.421C>A and imatinib plasma C_trough_ in GIST and CML patients.

## 1. Introduction

The tyrosine kinase inhibitor (TKI) imatinib mesylate is the treatment of choice for the management of chronic myeloid leukemia (CML) and gastrointestinal stromal tumors (GIST) [1,2] and the first small molecule to be introduced in oncology for targeted therapy.

Despite the outstanding efficacy of imatinib in CML and in GIST, a considerable portion of patients fails to achieve a stable therapeutic effect. It is estimated that approximately 30% of newly diagnosed CML and 10% of GIST patients develop resistance to imatinib within the first year of treatment, thus presenting an increased risk of experiencing poor survival outcomes [3,4].

Imatinib is a substrate of two efflux proteins, the P-glycoprotein (P-gP), encoded by the *multidrug resistance 1 (MDR1*, *ABCB1*) gene, and the breast cancer resistance protein (BCRP), encoded by the *ABCG2* gene. These proteins may play a dual role in the tumor resistance to imatinib. On one hand, the overexpression of P-gP and BCRP in tumor cells increases the tumor detoxification of the drug. On the other hand, as imatinib is a chronically assumed oral drug, P-gP and BCRP expression levels in normal tissue (*i.e*., intestinal lumen and biliary surface of hepatocytes) may affect its plasmatic concentration, potentially leading to under-exposure [5].

The *ABCB1* and *ABCG2* genes are highly polymorphic and some of the polymorphisms described were reported to affect the expression levels of the encoded proteins. Specifically, for *ABCB1*, the c.3435C>T (rs1045642), c.2677G>T/A (rs2032582), and c.1236C>T (rs1128503) polymorphisms were reported to lead to ABCB1 mRNA instability, thus leading to reduced protein expression and to the reduced functionality of P-gP in vivo [6,7]. For *ABCG2*, c.421C>A (rs2231142) is one of the most frequent polymorphisms and was found to be associated with lower protein expression in vitro and in vivo [8,9]. 

Several clinical investigations have highlighted the association between *ABCB1*/*ABCG2* polymorphisms and the efficacy of imatinib in both CML and GIST [10]. To what extent *ABCB1*/*ABCG2* polymorphisms contribute to the inter-individual variability in treatment efficacy by specifically affecting the imatinib pharmacokinetics has not been clarified. The few studies that have tried to address this point led to unclear findings, leaving unbridged the gap between *ABCB1*/*ABCG2* polymorphisms and imatinib exposure [11,12]. 

In the present study, we investigated the association between *ABCB1* c.3435C>T, c.2677G>T/A, c.1236C>T, ABCG2 c.421A>C, and the imatinib plasma trough concentration (C_trough_) in a prospective cohort of 33 GIST patients enrolled within a clinical trial at our Cancer Institute. To further substantiate our findings, we also carried out a meta-analysis of the published literature to quantitatively summarize the impact of *ABCB1* c.3435C>T, c.2677G>T/A, c.1236C>T, and *ABCG2* c.421A>C on imatinib plasma C_trough_ in CML and GIST. 

## 2. Results

### 2.1. Characteristics of the Patients

As of January 2022, 33 consecutive GIST patients, who met the inclusion criteria for the present study, were considered from those enrolled in the main clinical trial (2017-002437-369) [13]. Henceforth, the study population will be referred to as the CRO-Aviano Study. All patients were Caucasian. The detailed characteristics of the eligible patients are shown in Table 1.

### 2.2. CRO-Aviano Study

#### *ABC* Genotypes and Imatinib Trough Concentrations

Average imatinib C_trough_ in the 33 GIST patients included in the CRO-Aviano Study was 1040.6 ng/mL (IQR: 749.5–1292.4 ng/mL), ranging from a minimum of 255.8 ng/mL to a maximum of 2452.8 ng/mL. All 33 patients were successfully genotyped for the candidate variants and no significant deviation from the Hardy–Weinberg equilibrium (HWE) was observed for any of them. Detailed information on genotype and allele frequency distribution is reported in Table 2.

The comparison of imatinib C_trough_ according to *ABCB1* and *ABCG2* genotypes is shown in Table 3. No significant difference in imatinib plasma C_trough_ across different genotypes was observed.

Patients bearing the wild-type allele for all three *ABCB1* variants (c.3435C>T, c.2677G>T, and c.1236C>T) showed tendentially higher imatinib C_trough_ with respect to heterozygous or homozygous variant carriers. On the other hand, wild-type *ABCG2* c.421 CC carriers showed lower, although not significant, mean imatinib C_trough_ (1011.9 ng/mL) with respect to heterozygous (1030.3 ng/mL) and variant carriers (1792.8 ng/mL). The impact of the *ABCB1* c.3435C>T, c.2677G>T, c.1236C>T haplotype was assessed, but no significant difference was ascertained. 

### 2.3. Meta-Analysis

#### 2.3.1. Search Results and Study Characteristics

**Figure 1:**
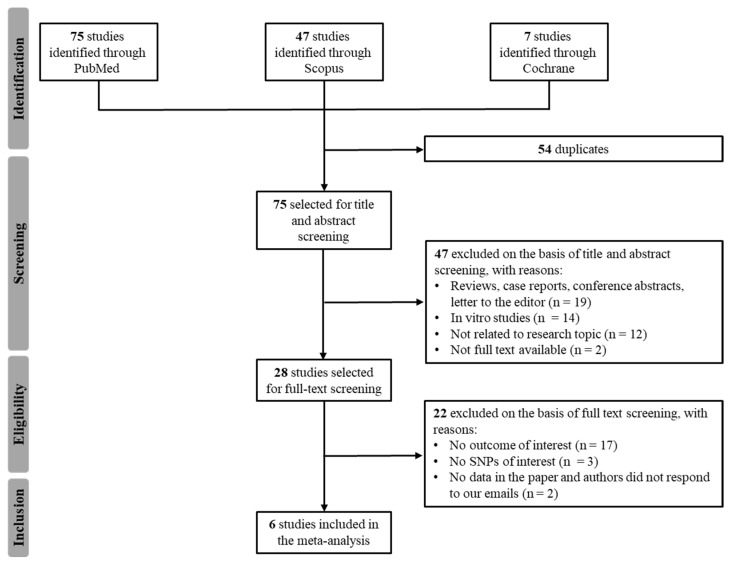
Flowchart of the literature review process.

A total of 75 potentially relevant articles were identified with our initial search strategy. After screening the titles and abstracts of articles, 47 studies were excluded because they were deemed repetitive or unqualified. After reading 28 potentially eligible papers, six studies from the literature met our inclusion criteria [14,15,16,17,18,19]. The study by Rajamani et al. did not report imatinib plasma C_trough_ for individual genotypes, but the authors provided the data on our request [14]. No additional studies were obtained by checking the reference lists of these articles. Figure 1 presents a detailed diagram of the above screening process. Our herein reported original CRO-Aviano Study was also included in the meta-analysis for a total of 7 studies.

The characteristics of the included studies are reported in Table 4. The sample size of the studies ranged from 33 to 173 patients, for a total of 649 patients. All studies included consecutive patients affected by GIST or CML. All seven studies (100%) analyzed the *ABCB1* c.3435C>T genotype, five (71.4%) studies analyzed the *ABCB1* c.1236C>T, four (57.1%) studies analyzed *ABCB1* c.2677G>T/A, and five (71.4%) studies analyzed *ABCG2* c.412C>A. The mean imatinib plasma C_trough_ ranged from 359.3 ng/mL to 2178.4 ng/mL. 

#### 2.3.2. Quality Assessment

All included studies reported satisfactory quality (MINORS score > 50.0%), with an average of 65.2% (range: 50.0%–87.5%), suggesting that the overall methodological quality of the included studies was from moderate to high (Table S1).

#### 2.3.3. Association between *ABCB1* c.3435C>T, c.2677G>T, and c.1236C>T and Imatinib C_trough_ Levels

A summary of random-effect models for the association between *ABCB1* c.3435C>T, c.2677G>T, c.1236C>T, and imatinib C_trough_ levels is reported in Table 5.

**Figure 2:**
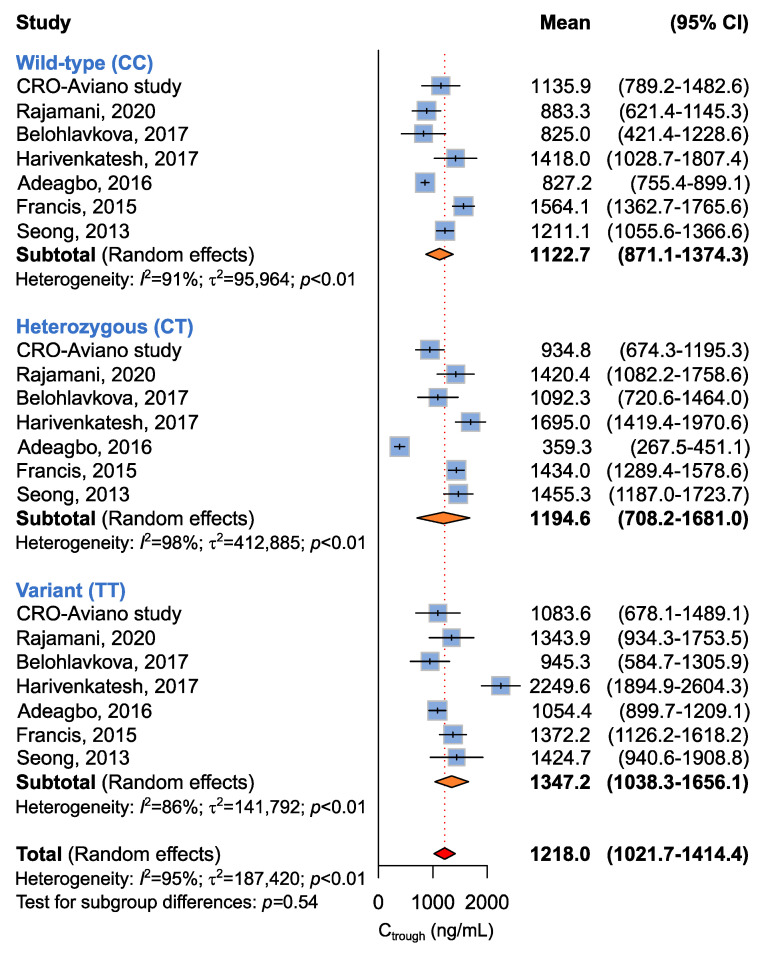
Forrest plots for the association between *ABCB1* c.3435C>T and imatinib C_trough_ levels for individual genotypes (allelic model) [14,15,16,17,18,19].

Seven studies including 649 patients administered with imatinib 400 mg per day were available for the association between *ABCB1* c.3435C>T and imatinib plasma C_trough_ levels [14,15,16,17,18,19], according to recessive (CC + CT vs. TT), dominant (CC vs. CT + TT), or additive (CC vs. CT vs. TT) models. The meta-analysis revealed no difference in imatinib plasma C_trough_ among different *ABCB1* c.3435C>T genotypes according to any model (Table 5 and Figure 2). However, significant heterogeneity among studies was found (*p* < 0.01).

**Figure 3:**
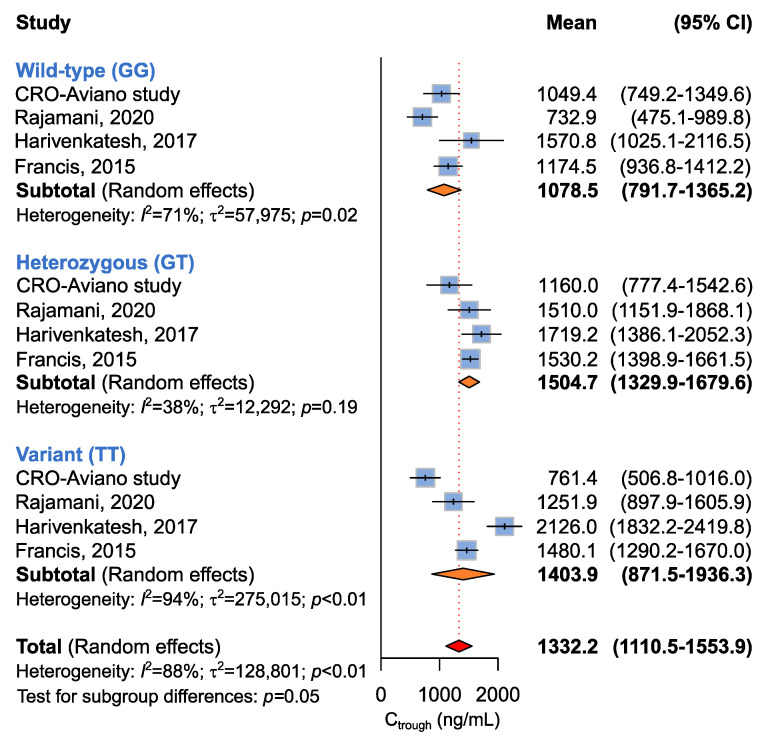
Forrest plots for the association between *ABCB1* c.2677G>T and imatinib C_trough_ levels for individual genotypes (allelic model) [14,17,18].

Four studies including 346 patients were available for the association between *ABCB1* c.2677G>T and imatinib plasma C_trough_ levels [14,17,18], according to recessive (GG + GT vs. TT), dominant (GG vs. GT + TT), or additive (GG vs. GT vs. TT) models. A borderline (*p* = 0.05) association between the *ABCB1* c.2677G>T genotype and imatinib plasma C_trough_ was highlighted by the additive model (Table 5 and Figure 3). Imatinib plasma C_trough_ was lower in *ABCB1* c.2677 GG carriers (C_trough_, 1078.5 ng/mL) with respect to the GT/A carriers (C_trough_,1504.7 ng/mL) and the TT/AA carriers (1403.9 ng/mL). The same trend was maintained when considering the dominant model, where homozygous carriers of the *ABCB1* c.2677 G allele showed lower imatinib C_trough_ when compared to the T/A allele carriers (C_trough_, 1078.5 ng/mL vs. 1475.9 ng/mL, *p* = 0.06) (Table 5). 

**Figure 4:**
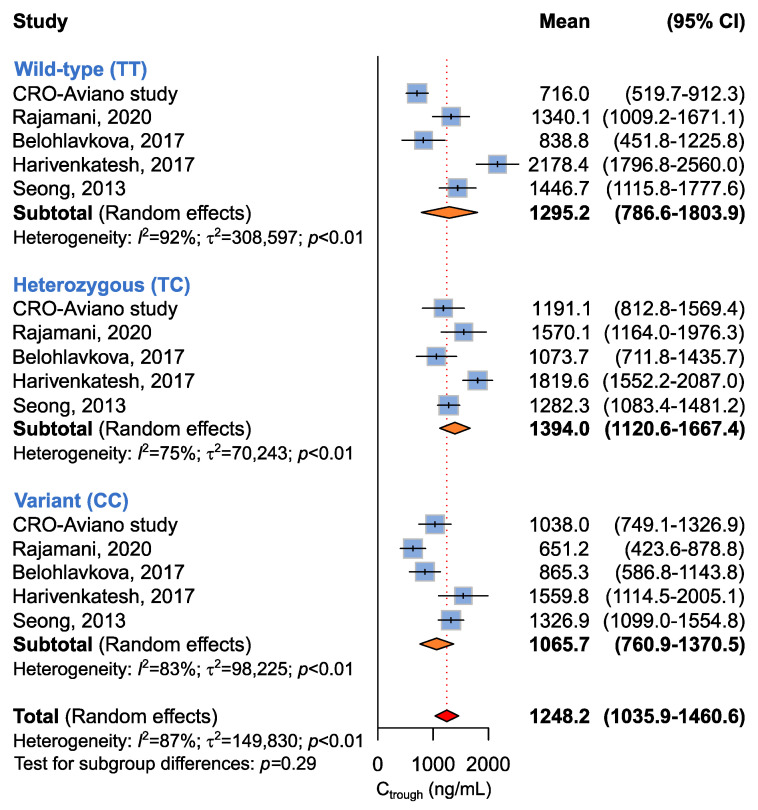
Forrest plots for the association between *ABCB1* c.1236C>T and imatinib C_trough_ levels for individual genotypes (allelic model) [14,16,18,19].

Five studies involving 467 patients were available for the association between *ABCB1* c.1236C>T and imatinib plasma C_trough_ [14,16,18,19], according to recessive (CC + CT vs. TT), dominant (CC vs. CT + TT), or additive (CC vs. CT vs. TT) models. The meta-analysis did not highlight any difference in imatinib plasma C_trough_ among *ABCB1* c.1236C>T genotypes according to any genetic model (Table 5 and Figure 4).

#### 2.3.4. Association between *ABCG2* c.412C>A and Imatinib C_trough_ Levels

**Figure 5:**
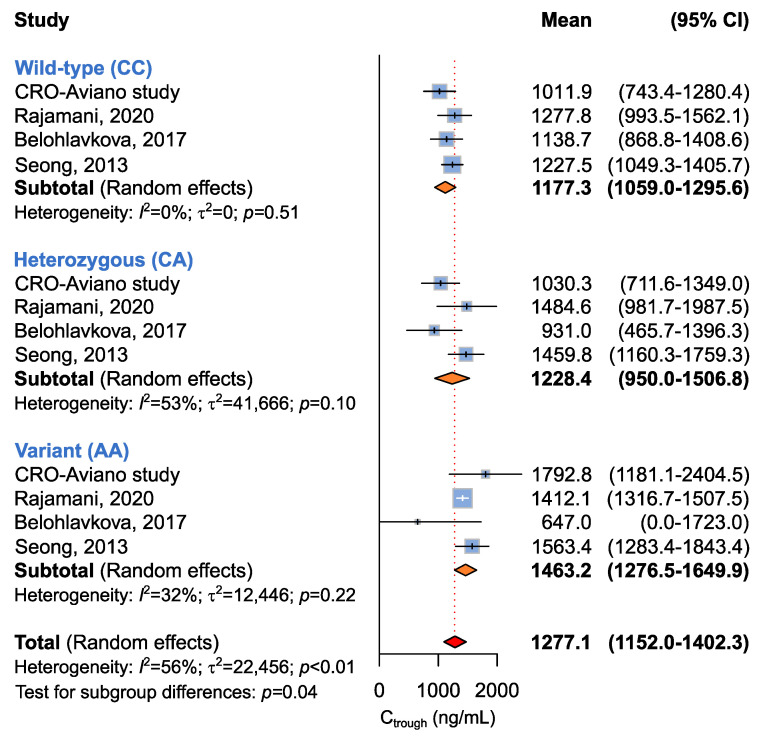
Forrest plots for the association between *ABCG2* c.412C>A and imatinib C_trough_ levels for individual genotypes (allelic model) [14,16,19].

A summary of random-effect models for the association between *ABCG2* c.412C>A and imatinib C_trough_ levels is reported in Table 5. Four studies involving 293 patients were available for the association between *ABCG2* c.412C>A and imatinib plasma C_trough_ [14,16,19]. The study from Francis et al. was not considered for this analysis, although the *ABCG2* c.412C>A variant was assessed, because no homozygous carriers of the A allele were detected [17]. The four included studies were utilized to evaluate the association between *ABCG2* c.412C>A and imatinib plasma C_trough_ according to recessive (CC + CA vs. AA), dominant (CC vs. CA + AA), or additive (CC vs. CA vs. AA) models. A significant difference in imatinib plasma C_trough_ was identified under the recessive (*p* = 0.04) and additive models (*p* = 0.04) (Table 5 and Figure 5). Under the recessive model, homozygous carriers of the A allele showed significantly higher imatinib plasma C_trough_ with respect to the CC/CA carriers (C_trough_, 1463.2 ng/mL vs. 1196.6 ng/mL). The additive model showed that CC carriers had mean imatinib C_trough_ (imatinib C_trough_ 1177.3 ng/mL) lower than the AC (imatinib C_trough_ 1228.4 ng/mL) and the AA carriers (imatinib C_trough_ 1463.2 ng/mL).

## 3. Discussion

Therapeutic failure in CML and GIST due to imatinib resistance is responsible for disease progression and shorter survival perspectives and remains an unmet challenge in clinical oncology. Addressing the complex phenomenon of imatinib resistance can help clinicians to personalize treatments by proposing imatinib dose adjustments or a switch to other therapies.

Inadequate exposure to imatinib is one of the leading mechanisms of treatment resistance. In this context, the activity of the efflux transporters P-gP and BCRP plays a pivotal role modulating the quantity of drug reaching the systemic circulation, and hence the tumor cell, as well as the amount of drug that is delivered to the intracellular molecular targets.

Thus far, most of the published literature has focused on the association between the *ABCB1/ABCG2* genotype and treatment clinical outcomes in CML and GIST [20,21,22,23]. In this regard, an imatinib C_trough_ threshold of 1100 ng/mL and 1000 ng/mL was proposed to guarantee a clinical benefit in GIST and CML, respectively [24,25]. Despite these preliminary indications on imatinib plasma titration, still little is known about the impact of *ABCB1/ABCG2* germline variants on imatinib systemic exposure as a key step to determine the development of resistance. Here, we investigated the impact of the most widely studied *ABCB1* c.3435C>T, c.2677G>T/A c.1236C>T, and *ABCG2* c.421C>A polymorphisms on the imatinib C_trough_ in a prospective cohort of 33 Caucasian GIST patients. To the best of our knowledge, this is the first time that the association between the *ABCB1/ABCG2* genotype and imatinib plasma C_trough_ has been assessed in GIST. Despite small differences in pharmacokinetic properties between GIST and CML patients, still some variability can be observed as a consequence of liver metastasis or gastric/intestinal surgery affecting GIST patients. Therefore, a thorough and dedicated investigation of factors affecting imatinib disposition in GIST is urgently needed to provide a rationale for dose adjustment [26]. To further substantiate our hypothesis, we next performed a meta-analysis on the published literature including studies that correlated the *ABCB1/ABCG2* genotype and imatinib plasma C_trough_ in GIST and CML.

In the CRO-Aviano Study, a trend was highlighted between the *ABCG2* c.421A allele and higher imatinib C_trough_ with respect to the C allele, which is concordant with the expected effect of the SNP on the protein, and this became statistically significant when meta-analyzed with another three eligible published studies. The studies included in the meta-analysis were small (up to 173 patients), not allowing sufficient statistical power when taken alone. Moreover, the heterogeneity of the *ABCG2* c.421C>A minor allele frequency (MAF) across different ethnical groups (around 0.10 in Europeans, 0.02 in Africans, 0.30 in Asians) can make less evident the effect of the genotype in studies involving a narrow number of *ABCG2* c.421 AA carriers. Nevertheless, the possibility to perform a meta-analysis allowed us to demonstrate the presence of a significant correlation between *ABCG2* c.421C>A and imatinib plasma C_trough_ in CML and GIST patients

BCRP is the protein encoded by *ABCG2* and is located on the apical surface of intestinal epithelia cells and on the biliary surface of hepatocytes, where it acts as an efflux transporter. The non-synonymous variant at the coding position 421 at exon 5, entailing a C>A nucleotide transition, is one of the most studied polymorphisms of *ABCG2* (rs2231142). The minor allele frequency (MAF) of the variant is equal to 10.3% in the European population, but it reaches 29.7% in the Asian population. The nucleotide switch results in a Gln to Lys substitution at the protein position 141 (Q141L), which has been associated with lower protein expression in breast cancer cell lines [8]. Intriguingly, Gardner et al. showed that cells expressing the BCRP protein with the 421AA genotype had significantly higher imatinib accumulation with respect to the wild-type protein [27], sustaining the defective extrusion of the drug in the presence of the variant allele. Consistently, Takahashi et al. observed that the dose-adjusted imatinib C_trough_ was higher in patients with the AA or CA genotype for *ABCG2* c.421C>A with respect to the CC carriers (dominant model) in a cohort of 67 Japanese CML patients [28]. Moreover, Petain et al. reported significantly lower imatinib clearance in heterozygous versus wild-type GIST patients for *ABCG2* c.421C>A (clearance: −23%; *p* < 0.05) [29]. In agreement with our findings, in a study involving 82 CML patients, Seong et al. [19] reported a non-significant trend between the *ABCG2* c.421A allele and higher imatinib C_trough_. Although some studies reported a lack of any possible association between *ABCG2* c.421C>A and imatinib C_trough_ [16,30], taken together, ours and previous reports suggest that the *ABCG2* c.421C>A genotype might play a key role in modulating imatinib disposition in GIST and CML.

More controversial are the results that we obtained both in our study cohort and in the successive meta-analysis on the role of *ABCB1* SNPs in imatinib pharmacokinetics. From our data, no significant effect of the *ABCB1* analyzed variants and imatinib plasma C_trough_ has emerged. The meta-analysis also failed to underscore a significant association between any of the *ABCB1* variants and the imatinib plasma C_trough_. Only patients who were heterozygous carriers of the variant T/A alleles for *ABCB1* c.2677G>T/A exhibited non-significantly higher imatinib C_trough_ with respect to the wild-type patients, under the additive (*p* = 0.05) or dominant (*p* = 0.06) models. It must be noted that the herein analyzed *ABCB1* polymorphisms, c.3435C>T, c.2677G>T/A, and c.1236C>T, are in strong linkage disequilibrium with each other and have been associated with reduced P-gP expression and functionality [7]. Of particular interest seems to be the *ABCB1* haplotype comprising the three c.3435C>T, c.2677G>T/A, and c.1236C>T variants. The presence of the minor allele in each genotype for the three *ABCB1* polymorphisms was previously associated with lower P-gP activity with respect to the wild-type *ABCB1* and with a lower imatinib export capacity [7,31].

The lack of information on each single *ABCB1* polymorphism in the meta-analyzed studies prevented us from investigating the impact of the *ABCB1* TTT haplotype on imatinib plasma C_trough_, and the single SNP approach could have hindered the actual impact of the *ABCB1* haplotype. 

The impact of the *ABCB1* genotype on imatinib disposition and pharmacokinetics showed conflicting results in the published literature, leaving plenty of room for further devoted investigations [10]. It is also worth considering that P-gP is a common player in drug–drug interactions [27,32], and that its expression can be modulated by the concurrent intake of P-gP inducer or inhibitor agents. Together with the genotype, the concurrent effect of interacting drugs on P-gP activity can further complicate the prediction of its effect on the pharmacokinetics of oral drugs and can partially explain the lack of consistency of the published literature on imatinib C_trough_. Due to the small size of our study cohort, as well as the complex phenomenon of drug–drug and drug–gene interaction, the potential effect of concomitant interacting drugs on imatinib C_trough_ was not considered in the present study and remains an issue to be investigated in thorough, dedicated studies. 

Some limitations of the present study must be acknowledged. First, the small number as well as the small sample sizes of included studies might have obscured our findings. Second, the heterogeneity of ethnic groups and the different analytical methods used to quantify imatinib plasma concentrations could have contributed to the wide inter-study heterogeneity here observed. In addition, the impossibility of retrieving individual imatinib C_trough_ and genotypes from most of the corresponding authors prevented us from conducting a pooled analysis on raw data. However, although the impact of *ABCB1* on imatinib disposition remains to be further addressed, a clear effect of *ABCG2* c.421C>A on imatinib exposure has emerged, suggesting that the genotyping of *ABCG2* c.421C>A may be a valuable tool to explain the variability in imatinib pharmacokinetics. As a future perspective, joint collaborations including multiple research groups focused on the present topic to pool data on imatinib C_trough_ and the *ABCB1/ABCG2* genotype will help to shed light on the actual contribution of transporters’ pharmacogenetics to imatinib disposition.

In summary, the available studies and our original results presented here support a consistent correlation between *ABCG2* c.421C>A and imatinib plasma C_trough_. The current meta-analysis provides evidence that CML and GIST patients administered with imatinib carrying the homozygous variant of *ABCG2* c.421C>A show higher imatinib C_trough_ with respect to wild-type and heterozygous patients. This finding indicates the need for a thorough, extensive characterization of genetic variants affecting imatinib transporters in wider study populations and supports the implementation of pharmacogenetics to refine imatinib dose adjustment. 

## 4. Materials and Methods

### 4.1. Ethics Statements

Patients who were enrolled at IRCCS Centro di Riferimento Oncologico di Aviano (Italy) within a prospective clinical trial (EudraCT number 2017-002437-36) provided signed informed consent at the time of enrollment. The research protocol was registered by AIFA and was approved by the Local Ethical Committee as previously described [13]. All analyses and data handling were conducted according to the Declaration of Helsinki. 

### 4.2. Patient Selection

The clinical research protocol had the primary aim to assess the feasibility of the routine therapeutic drug monitoring (TDM) of imatinib and circulating tumor DNA analysis in serial blood samples from GIST patients. Eligibility criteria were as follows: (i) histologically confirmed GIST; (ii) treatment with imatinib > 90 days prior to study entry, regardless of the administration setting; (iii) Eastern Cooperative Oncology Group (ECOG) performance status of 0 or 1; (iv) adequate liver, renal and bone marrow function; (v) age ≥ 18; (vi) capability of attending scheduled medical check-ups regularly; (vii) signed informed consent. 

For the purposes of the herein presented sub-study, patients who met specific additional requirements were selected: (i) administration of imatinib 400 mg daily dose, (ii) concentration of imatinib above the lower limit of quantification (LLOQ; i.e., 30 ng/mL imatinib).

### 4.3. Blood Collection and Genotyping 

First, 15 mL of blood was routinely collected as a per-protocol methodology in K_2_-EDTA containing tubes at the time of regular medical check-ups, every 3 to 6 months. Plasma was separated by means of centrifugation and stored at −80 °C until imatinib quantification. Genomic DNA was extracted from the harvested buffy coat by means of the GeneJET Whole Blood Genomic DNA Purification Mini Kit (Thermo Fisher Scientific, Wilmington, DE, USA) and quantified with a Quantus Fluorometer (Promega, Madison, WI, USA). Genomic DNA was stored at 4 °C. *ABCB1* and *ABCG2* were analyzed by means of targeted next-generation sequencing (NGS). Sequencing libraries were prepared starting from 100 ng of genomic DNA using a custom hybridization-based NimbleGen SeqCap EZ Choice Library (Roche, Inc., Madison, WI, USA) targeting the UTRs and the coding sequences of 60 cancer-related genes, according to the NimbleGen SeqCap EZ Library SR User’s Guide v3.0 (Roche, Inc. Madison, WI, USA). Genomic DNA was enzymatically fragmented for 15 min at 37 °C, end-repaired, A-tailed and ligated with Illumina-indexed adapters. Ligated libraries were size-selected by means of Agencourt AMPure XP Beads (Beckman Coulter, Brea, CA, USA) to retain fragments ranging between 300 and 350 bp and amplified in 12 PCR cycles. Final libraries were quantified by means of the Quantus Fluorometer (Promega, Madison, WI, USA) with the QuantiFluor dsDNA Dye (Promega, Madison, WI, USA) and the fragment size analysis was performed using the Agilent 4200 TapeStation (Agilent Technologies, Santa Clara, CA, USA) with High-Sensitivity D1000 ScreenTape. Pooled libraries were obtained by putting together 45 ng of each sample that was amplified in the regions of interest by using the SeqCap EZ Choice Library (Roche, Inc., Madison, WI, USA), followed by 7 PCR cycles. At the time of sequencing, libraries were denatured with fresh NaOH 0.2 M and diluted to a final concentration of 10 pM. Generated FASTQ files were processed using the Variant Studio software (Illumina, San Diego, CA, USA), for variants’ annotation. 

### 4.4. Imatinib Quantification

Quantification of imatinib was performed using an LC-MS/MS instrument consisting of a Prominence LC-20AD UFLC XR (Shimadzu, Tokyo, Japan) and an API 4000 QTrap mass spectrometer (SCIEX, Framingham, MA, USA). Imatinib was quantified after protein precipitation by means of a methanol-based extraction method. Imatinib was separated on a Synergy Fusion RP C18 chromatography column 4 μm, 50 × 2.0 mm, coupled to a C18 precolumn (Phenomenex, Torrence, CA, USA). Elution was performed in gradient-mode chromatography. The mass spectrometer was equipped with an electrospray ionization (ESI) source interface operating in positive ion mode. The biological samples were analyzed in Selected Reaction Monitoring mode. Quantifications were performed using the following transitions: *m/z* 494.4 > 394.2 for imatinib and *m/z* 502.4 > 394.2 for imatinib-D8, used as an internal standard. The developed method was validated according to FDA and EMA guidelines for the validation of bioanalytical methods, evaluating the linearity, recovery, limit of detection, limit of quantification, matrix effect, inter- and intra-day precision and accuracy, selectivity, stability and reproducibility. To guarantee the homogeneous quantification of imatinib C_trough_, blood samples were possibly collected 24 h after the last imatinib administration. If imatinib had not been administered exactly 24 h prior to blood collection, the following formula, previously validated by Wang et al. [33], was used to extrapolate imatinib C_trough_:

(1) $$C_{trough}=C*0.5^{\frac{24-T}{T_{1/2}}}$$

*C* = measured drug concentration, *T* = hours from the last drug administration, *T*_1/2_ = drug plasma half-life (18 h).

The samples collected up to five hours or more than thirty-five hours from the last imatinib administration were excluded from the analysis as they were outside the algorithm’s range of linearity.

### 4.5. Meta-Analysis

#### 4.5.1. Search Strategy

The meta-analysis was conducted following the Preferred Reporting Items for Systematic Reviews and Meta-Analyses (PRISMA) checklist [34]. PubMed, Scopus and Cochrane were searched from November 2021 to April 2022. The following keywords were used: “imatinib” OR “imatinib mesylate” OR “Gleevec” AND “plasma exposure” OR “Ctrough” OR “trough concentration” OR “trough levels” OR “Cmin” OR “minimum concentration” OR “minimum levels” AND “*ABCB1*” OR “*ABCG2*” OR “transporters” OR “pharmacogenetics”. No time restrictions were applied. Two authors (CDF and EDM) independently screened all titles and abstracts generated by the search and then evaluated the full texts of relevant articles identified against the inclusion criteria (Figure 1). A third author (EC) settled discordances when present. 

#### 4.5.2. Selection Criteria

Studies were included in the analysis if they met the following criteria: (i) the study included patients administered imatinib for more than one month for the management of GIST or CML; (ii) patients were treated with an imatinib standard dose of 400 mg/die; (iii) studies reported imatinib plasma C_trough_ at the steady state; (iv) studies reported the association between individual polymorphisms in *ABCB1* and/or *ABCG2* and imatinib plasma C_trough_. Non-English studies were excluded. Studies containing duplicated data from previously published works were excluded, as well as review articles, case reports, editorials and letters. Two authors (CDF, EDM) independently assessed the quality of the included studies according to the Methodological Index for Non-Randomized Studies (MINORS) criteria [35]. MINORS consists of a validated, 12-item scoring system for non-randomized studies, with a global ideal score of 16 for non-comparative studies and 24 for comparative studies. For each item, the MINORS scale assigns scores of 0 (not reported), 1 (reported but inadequate) and 2 (reported and adequate). The MINORS score was reported as a percentage of the global ideal score. Low-quality (score < 50.0%) studies were excluded from the meta-analysis. 

#### 4.5.3. Statistical Analysis 

Clinical data were presented as absolute frequencies and percentages and as median and range as appropriate. Chi-squared analysis was used to test for genotype deviation from Hardy–Weinberg equilibrium. In the patients included in the CRO-Aviano Study, the association between *ABCB1*/*ABCG2* genotypes and imatinib plasma C_trough_ was assessed by the non-parametric Kruskal–Wallis test. Mean imatinib plasma C_trough_ and corresponding standard deviation by genotype to be used in the meta-analysis were retrieved from selected articles or our present case series. Dominant and recessive models were evaluated combining mean imatinib plasma C_trough_ and corresponding standard deviation of heterozygous or variant carriers for the former, and of wild-type and heterozygous carriers for the latter. The pooled mean imatinib plasma C_trough_ and corresponding 95% confidence interval (CI) were calculated according to random-effects models of DerSimonian and Laird; statistical heterogeneity among studies was evaluated using the *I*² and τ^2^ statistics [36]. The results of the meta-analysis were presented graphically using Forrest plots, plotting the individual papers and mean C_trough_ with corresponding 95% CI. Statistical significance was claimed for *p* < 0.05 (two-sided). 

## Figures and Tables

**Table 1:** Demographic and clinical characteristics of 33 GIST patients included in the CRO-Aviano Study.

Patient Characteristic	N	%
Gender		
Male	16	48.5
Female	17	51.5
Age at enrollment		
Median (range)	66 (35–83)	
Primary tumor site		
Stomach	15	45.5
Intestinal	13	39.4
Other ^1^	5	15.1
Imatinib setting at enrollment		
Adjuvant	9	27.3
First line	24	72.7

^1^ Abdominal, pelvic region and peritoneum.

**Table 2:** Genotype and allele frequency distribution for *ABCB1* c.3435C>T, c.2677G>T, c.1236C>T, and *ABCG2* c.412C>A in CRO-Aviano Study.

SNP	Genotype Frequency, N (%)			Allele Frequency		HWE
	Wild Type	Het	Variant	*p*	*q*	*p*-Value
*ABCB1* c.3435C>T	9 (27.3)	15 (45.4)	9 (27.3)	0.500	0.500	0.3657
*ABCB1* c.2677G>T	14 (42.4)	13 (39.4)	6 (18.2)	0.621	0.379	0.1050
*ABCB1* c.1236C>T	14 (42.4)	13 (39.4)	6 (18.2)	0.621	0.379	0.1050
*ABCG2* c.412C>A	23 (69.7)	9 (27.7)	1 (3.0)	0.836	0.164	0.8564

**Table 3:** Comparison of imatinib C_trough_ with the transporter genotype in CRO-Aviano Study.

Genotype	N	Imatinib C_trough_		*p*-Value
		Mean	SD	
*ABCB1* c.3435C>T				
Wild-type	9	1135.9	530.7	0.4186
Heterozygous	15	934.8	514.7	
Variant	9	1083.6	620.7	
*ABCB1* c.2677G>T				
Wild-type	14	1049.4	573.1	0.1907
Heterozygous	13	1160.0	703.8	
Variant	6	761.4	318.2	
*ABCB1* c.1236C>T				
Wild-type	14	1038.0	551.6	0.1907
Heterozygous	13	1191.1	695.9	
Variant	6	716.0	245.3	
*ABCB1* Haplotype				
Wild-type	8	1135.9	412.6	0.2185
Heterozygous	20	1059.3	566.0	
Variant	5	813.5	279.7	
*ABCG2* c.412C>A				
Wild-type	23	1011.9	657.0	0.5434
Heterozygous	9	1030.3	487.8	
Variant	1	1792.8	-	

**Table 4:** Characteristics of the studies selected for the meta-analysis.

Study	Country	Disease	Study Type	Enrollment Interval	Sample Size (Genotyped)	Genotyping Method	Imatinib Quantification Method	Analyzed SNPs	Wild-Type (n)	Heterozygous (n)	Variant (n)
Adeagbo et al. [15]	Nigeria	CML	Observational, case–control, prospective	N.A.	110 (109)	TaqMan allele discrimination assay	HPLC coupled with a diode array UV detector	*ABCB1* c.3435C>T	80	24	5
Belohlavkova et al. [16]	Czech Republic	CML	Observational, retrospective	1997–2012	112	TaqMan allele discrimination assay	HPLC	*ABCB1* c.3435C>T	22	58	32
								*ABCB1* c.1236C>T	32	62	18
								*ABCG2* c.421C>A	87	23	2
Francis et al. [17]	India	CML	Observational, prospective	2012–2014	111 (73)	TaqMan allele discrimination assay	HPLC coupled with electrospray-ionization MS/MS	*ABCB1* c.3435C>T	11	37	25
								*ABCB1* c.2677G>T	8	33	32
								*ABCG2* c.421C>A	54	19	0
Harivenkatesh et al. [18]	India	CML	Observational, prospective	2013–2016	173	Direct Sanger sequencing	HPLC coupled with MS/MS	*ABCB1* c.3435C>T	19	88	66
								*ABCB1* c.1236C>T	19	113	41
								*ABCB1* c.2677G>T	18	76	79
Rajamani et al. [14]	India	CML	Observational, prospective	N.A.	160 (67)	PCR restriction fragment length polymorphism (RFLP)	HPLC	*ABCB1* c.3435C>T	7	39	21
								*ABCB1* c.1236C>T	10	30	27
								*ABCB1* c.2677G>T	6	35	26
								*ABCG2* c.421C>A	45	17	5
Seong et al. [19]	Korea	CML	Observational, prospective	N.A.	82	TaqMan allele discrimination assay	HPLC coupled with MS/MS	*ABCB1* c.3435C>T	35	38	9
								*ABCB1* c.1236C>T	17	37	28
								*ABCG2* c.421C>A	41	32	8
CRO-Aviano Study	Italy	GIST	Observational, prospective	2015–2021	33	Targeted NGS	HPLC coupled with MS/MS	*ABCB1* c.3435C>T	9	15	9
								*ABCB1* c.1236C>T	14	13	6
								*ABCB1* c.2677G>T	14	13	6
								*ABCG2* c.421C>A	23	9	1

**Table 5:** Summary of random effect meta-analyses for *ABCB1* c.3435C>T, c.2677G>T/A, c.1236C>T, and *ABCG2* c.421C>T.

Genotype	N Studies	C_trough_	Test for Heterogeneity		Subgroup Differences
	Studies (Total Patients)	Mean (95% CI)	*I*^2^ %	*p*-Value	*p*-Value
*ABCB1* c.3435C>T					
CC + CT vs. TT ^a^	7 (649)	1209.8 (893.6–1526.1) vs. 1378.6 (1059.3–1698.0)	95	<0.01	0.46
CC vs. CT + TT ^b^		1088.1 (876.7–1297.5) vs. 1250.9 (790.6–1711.1)	96	<0.01	0.53
CC vs. CT vs. TT ^c^		1122.7 (871.1–1374.3) vs. 1194.6 (708.2–1681.0) vs. 1347.0 (1038.3–1656.1)	95	<0.01	0.54
*ABCB1* c.2677G>T					
GG + GT vs. TT ^a^	4 (346)	1408.7 (1196.1–1621.4) vs. 1403.0 (871.0–1936.3)	88	<0.01	0.99
GG vs. GT + TT ^b^		1078.5 (791.7–1364.2) vs. 1475.9 (1174.2–1777.7)	89	<0.01	0.06
GG vs. GT vs. TT ^c^		1078.5 (791.7–1365.2) vs. 1504.7 (1329.9–1679.6) vs. 1403.9 (871.5–1936.3)	88	<0.01	0.05
*ABCB1* c.1236C>T					
CC + CT vs. TT ^a^	5 (467)	1307.3 (1057.7–1556.9) vs. 1295.2 (786.6–1803.9)	89	<0.01	0.97
CC vs. CT + TT ^b^		1065.7 (760.9–1370.5) vs. 1365.4 (1049.3–1681.4)	89	<0.01	0.18
CC vs. CT vs. TT ^c^		1065.7 (760.9–1370.5) vs. 1394.0 (1120.6–1667.4) vs. 1295.2 (786.6–1803.9)	87	<0.01	0.29
*ABCG2* c.412C>A					
CC + CA vs. AA ^a^	4 (293)	1198.6 (1036.0–1361.2) vs. 1463.2 (1276.5–1649.9)	67	<0.01	0.04
CC vs. CA + AA ^b^		1177.3 (1059.0–1295.6) vs. 1263.47 (1000.6–1526.3)	37	0.14	0.56
CC vs. CA vs. AA ^c^		1177.3 (1059.0–1295.6) vs. 1228.4 (950.01–1506.8) vs. 1463.2 (1276.5–1649.9)	56	<0.01	0.04

^a^ Recessive model. ^b^ Dominant model. ^c^ Additive model.

## Data Availability

Data available on request from the authors. The data that support the findings of this study are available from the corresponding author upon reasonable request. Some data may not be made available because of privacy or ethical restrictions.

## Supplementary Materials

The following supporting information can be downloaded at: https://www.mdpi.com/article/10.3390/ijms24043303/s1.
