# Supplementary material for: Impact of ABCG2 and ABCB1 Polymorphisms on Imatinib Plasmatic Exposure: An Original Work and Meta-Analysis
Source: Int J Mol Sci. 2023 Feb 7;24(4):3303. doi: 10.3390/ijms24043303 (PMC9963452; doi:10.3390/ijms24043303)
Supplement: Supplementary file 1 [file ijms-24-03303-s001.zip › ijms-2042448-supplementary.pdf]

Table S1. Quality assessment of included studies

Criteria	Adeagbo et al .[15]	Belohlavkova et al. [16]	Francis et al. [17]	Harivenkatesh et al. [18]	Rajamani et al. [14]	Seong et al. [19]	CRO-Aviano Study
**1**. A clearly stated aim	2	2	2	2	2	2	2
**2**. Inclusion of consecutive patients	0	0	0	2	2	0	2
**3**. Prospective collection of data	0	0	0	2	2	0	2
**4**. Endpoints appropriate to the aim of the study	1	2	2	1	2	1	2
**5**. Unbiased assessment of the study end-point	2	2	2	2	2	2	2
**6**. Follow-up period appropriate to the aim of the study	2	2	2	2	2	2	2
**7**. Loss to follow up less than 5%	1	0	0	2	2	1	2
**8**. Prospective calculation of the study size	0	0	0	0	0	0	0
**9**. An adequate control group	NA	NA	NA	NA	NA	NA	NA
**10**. Contemporary groups	NA	NA	NA	NA	NA	NA	NA
**11**. Baseline equivalence of groups	NA	NA	NA	NA	NA	NA	NA
**12**. Adequate statistical analyses	NA	NA	NA	NA	NA	NA	NA
**Total MINORS score**	**8**	**8**	**8**	**13**	**14**	**8**	**14**
**Maximum possible score**	**16**	**16**	**16**	**16**	**16**	**16**	**16**
**MINORS score expressed as % of the maximum possible score**	**50.0%**	**50.0%**	**50.0%**	**81.3%**	**87.5%**	**50.0%**	**87.5%**
